# What Is Citizen Science? – A Scientometric Meta-Analysis

**DOI:** 10.1371/journal.pone.0147152

**Published:** 2016-01-14

**Authors:** Christopher Kullenberg, Dick Kasperowski

**Affiliations:** Department of Philosophy, Linguistics and Theory of Science, Univerisity Gothenburg, Gothenburg, Sweden; Universidad de Las Palmas de Gran Canaria, SPAIN

## Abstract

**Context:**

The concept of citizen science (CS) is currently referred to by many actors inside and outside science and research. Several descriptions of this purportedly new approach of science are often heard in connection with large datasets and the possibilities of mobilizing crowds outside science to assists with observations and classifications. However, other accounts refer to CS as a way of democratizing science, aiding concerned communities in creating data to influence policy and as a way of promoting political decision processes involving environment and health.

**Objective:**

In this study we analyse two datasets (N = 1935, N = 633) retrieved from the Web of Science (WoS) with the aim of giving a scientometric description of what the concept of CS entails. We account for its development over time, and what strands of research that has adopted CS and give an assessment of what scientific output has been achieved in CS-related projects. To attain this, scientometric methods have been combined with qualitative approaches to render more precise search terms.

**Results:**

Results indicate that there are three main focal points of CS. The largest is composed of research on biology, conservation and ecology, and utilizes CS mainly as a methodology of collecting and classifying data. A second strand of research has emerged through geographic information research, where citizens participate in the collection of geographic data. Thirdly, there is a line of research relating to the social sciences and epidemiology, which studies and facilitates public participation in relation to environmental issues and health. In terms of scientific output, the largest body of articles are to be found in biology and conservation research. In absolute numbers, the amount of publications generated by CS is low (N = 1935), but over the past decade a new and very productive line of CS based on digital platforms has emerged for the collection and classification of data.

## Introduction

During the past decade “citizen science” (CS) has engaged an increasing number of academic researchers. The lion’s share of these well-circulated accounts tell a very favourable story of the successful involvement of non-scientists in research [1–6], even though concerns have been raised over data quality and the possibility of conflicts of interest among volunteers [7].

However, there are numerous instances in which volunteer contributors have remained invisible. In a recent study by Cooper et al. [8], the authors show that in research on climate change based on observations of avian migration, the contribution of data by citizen scientists amounted to almost 50 per cent in performed studies, even though proper credit was, in many cases, absent. Along similar lines, Silvertown claims that “hundreds” of scientific papers have relied on volunteer contributions in the past, without satisfactory recognition [1,9]. Cooper et al. conclude that one solution to such a problem of invisibility would be to adhere to a common usage of the keyword “citizen science” for each article based on data generated by non-scientists. But this suggestion is confronted with another problem, which is related to the diverse terminology describing volunteer contributions to science. The meaning of “citizen science” is in fact not very clear, particularly when formulated on a science policy level, where it is often defined too broadly without making the distinctions that scientists work with. A recent study by Follett and Strezov used an European Union policy definition for narrowing down their search result from using the term “citizen science” in the Scopus and Web of Science databases, while stressing that the term does not encompass the full breadth of projects involving volunteers from the general public [10]. Hence, the problem discussed by Cooper et al. is not only a historical problem for identifying CS literature, it is also a contemporary problem, which urgently needs to be worked around to get the richest possible description of research performed in this vein. In this article we use both qualitative and quantitative (scientometric) approaches in an attempt to remedy this potential source of confusion and limitation, to create a more useful map based on scientists’ own accounts of what CS entails. Consequently, we will have to go beyond the term CS to include also research found outside this narrow terminology.

## Objectives

Given the multiple descriptions of CS, the purpose of this article is to provide an insight into what CS is, with regards to the following questions:

RQ 1. How has CS and related terms developed over time?
RQ 2. What strands of research have adopted CS?
RQ 3. Which CS projects have a scientific output?

These questions will be answered with two datasets retrieved from the WoS Core Collection. One is based on a qualitative survey of relevant CS-related terminology combined with a set of recursive searches to find more and relevant search terms, and one is based on the names of individual citizen science projects, which have been retrieved from previous studies. This way, we create a dual approach for describing the phenomenon of CS. The paper then concludes with a discussion of the different strands of research that we have mapped out and a reflection upon the limitations of the scientometric method.

## Materials and Methods

### Identifying search terms related to “citizen science”

Perhaps the most elusive problem in describing CS originates from the multiple meanings of the concept itself. On a qualitative level this is evident by observing how two distinct meanings have developed in the natural- and social sciences respectively since the early to mid-1990s.

The most common conception of the meaning of CS, which in recent years has gained significant momentum in the natural sciences, originates in the type of research described by Bonney et al. who attest that “[i]n the past two decades, CLO’s [Cornell Laboratory of Ornithology] projects have engaged thousands of individuals in collecting and submitting data on bird observations” (p. 977). This practice, however, goes back at least to the 1960s and is sometimes even extended to include the National Audubon Society’s annual Christmas Bird Count, beginning in the year 1900, even if the name “citizen science” was not used until the 1990s [4]. In this line of research, the focal point for volunteer contributions consists of participation in observations, classification and collection of data, which in turn are used by scientists. There are important synonyms to the concept of CS in this case, including ‘community-based monitoring’ [3], ‘volunteer monitoring’ [11] and ‘participatory science’ [12], all designating the contribution of non-scientists to (primarily natural-) science.

On the other hand we find a very influential notion originating in the social sciences, as expressed in the account of Irwin’s 1995 book “*Citizen Science*: *A Study of People*, *Expertise and Sustainable Development*” where CS is defined as “/…/ a science which assists the needs and concerns of citizens /…/ a form of science developed and enacted by citizens themselves”[13]. This vision and approach has been widely adopted in the social sciences and by policy-makers, but it also describes and envisions research on health, such as in “popular epidemiology” [14]. In practice this conception of CS is understood largely as the roles of citizens as stakeholders in processes of scientifically informed decision-making.

These two major understandings do not, however, exhaust all forms of CS that are of relevance for researchers interested in this phenomenon. There is also a plethora of concepts that have been coined to describe primarily local and activist-oriented forms of CS. These are more difficult to trace via scientometric methods because the results are not published in peer-reviewed literature. Instead the data from these studies are mainly used for direct interventions in policy-making and litigations. However, such interventions are often made visible by social scientists doing research on the phenomenon of CS. For example, there are cases of activist-oriented CS were data are scientifically validated and used for legal action against polluting industries [15,16], Geographical Information Systems research for promoting the rights of indigenous peoples [17] and ‘civic technoscience’ for developing affordable instruments that can be used for monitoring oil spills and green urban areas [18]. Here we also find terms such as ‘community based auditing’ [19], ‘civic science’ [20] and ‘community environmental policing’ [16], ‘street science’ [21] and ‘popular epidemiology’ [14,22,23]. Finally, there are examples of organizational studies of ‘crowd science’ [24] and policy documents describing CS as ‘Do It Yourself Science’ [25]. These, however, only make sporadic appearances in the scientific literature.

### Recursive searches

A qualitative review of search terms for describing CS has clear limitations, the most obvious one being that false negatives will appear if a term is unknown to the researcher when constructing the search string. While false positives can be easily omitted if they are not too numerous, false negatives are much harder to detect. To minimize this problem we have conducted triple recursive searches (3RS) as documented in S1 Appendix. We conducted an initial search on 2015-10-13 based on a search string derived from the concepts described above. This resulted in 1281 records in the WoS Core Collection database. From these, we extracted all author keywords occurring more than three times (the DE field in the WoS file format). Then we applied the following inclusion- and exclusion criteria to generate a new recursive iteration of relevant search terms. To qualify as a new search term each had to represent some form active participation among non-scientists and had to be concerned with science, research or science policy. Thus, we excluded keywords that were:

1. Not exclusive to science. For example “crowdsourcing” and “public participation”.
2. Overlapping with existing search terms. For example “PPGIS” and “participatory GIS”.
3. Already included in the second dataset based on individual project names. For example “Ebird”.
4. Do not designate active participation of volunteers. For example “ranger-based monitoring” and “volunteers in research” were excluded because in the former case research is conducted by professionals and in the latter case volunteers are merely objects of study, not actively participating in scientific work.

On 2015-12-15 we performed three “snowball searches” to recursively include more search terms according to the above mentioned criteria. Thus we added “public engagement”, “participatory monitoring”, “participatory sensing”, “public participation in scientific research”, “locally based monitoring” and “volunteer based monitoring” to our original search string. This produced an additional 654 records, making the total N = 1935. As comparison, we used the same search string in the Scopus database, which returned 1954 records. Due to the complexity in comparing these two databases (search engine configuration, search algorithms, etc.) we decided to only use the WoS results in the present study.

### Quantifying search terms

**Fig 1:**
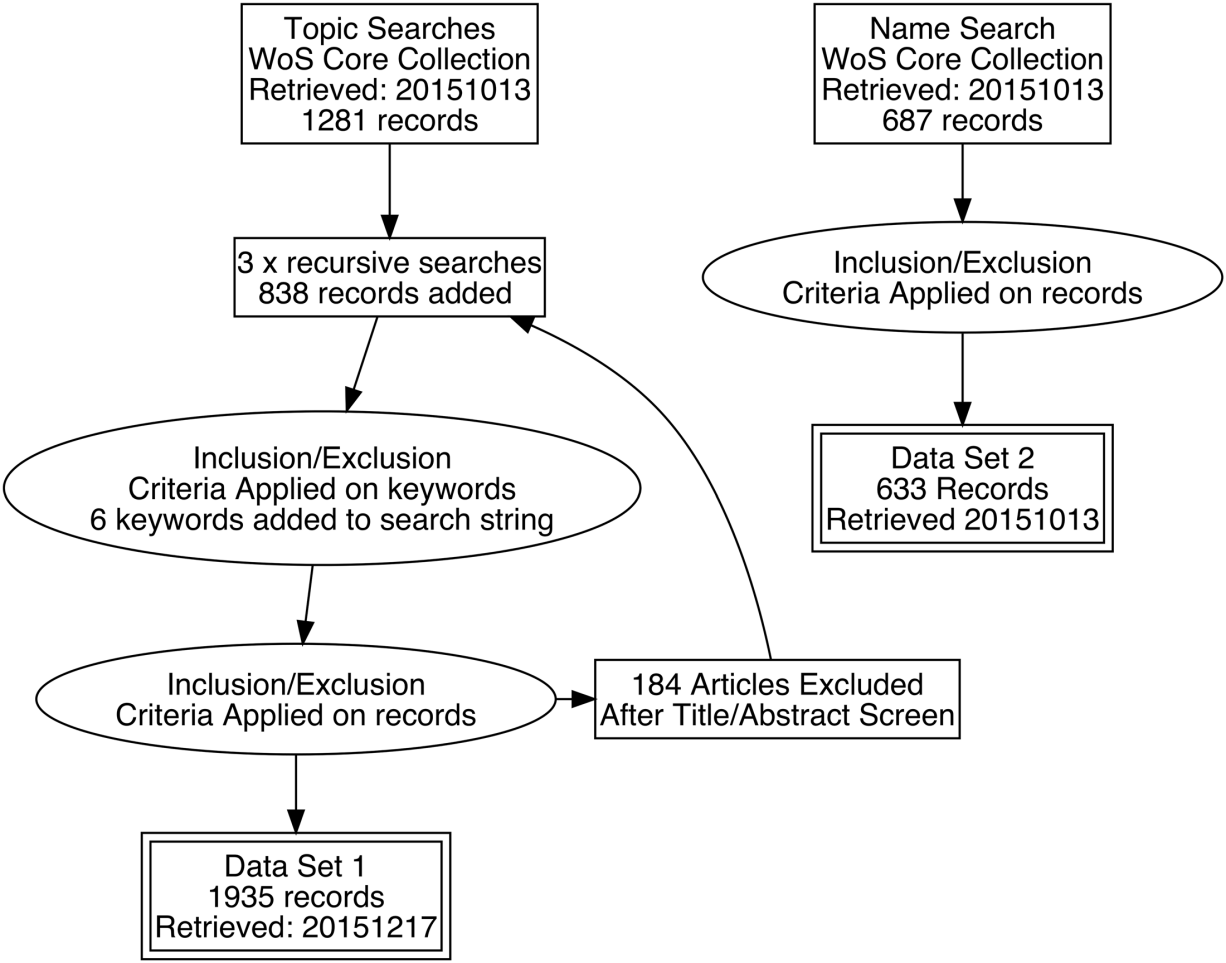
Selection procedure. Exact search strings and excluded search terms are listed in S1 Appendix.

Based on the qualitative survey combined with recursive searches, we retrieved 1935 articles from the Web of Science Core Collection using a search string composed of the terminology from the qualitative survey (S1 Appendix). The search was conducted on 2015-12-17. The search string was then manually checked against each article abstract in order to verify that the content of the article was relevant for inclusion. For example, the term “Community-based monitoring” is used to describe certain medical surveys that are unrelated to CS, thus being excluded since they lack active participation from volunteers. Also, we used regular expressions to search for semantic anomalies that appear in rare cases because of the inclusiveness of the Web of Science search engine. For example, a number of false positives appear when “citizen: science” or “citizen/science” are used in the abstracts or titles of the articles. A third source of false positives are generated by the WoS Keyword Plus [26], which are computer-generated keywords that are included in a Topic Search (TS). Because these are generated from the bibliographies of the articles, they are not always consistent with the overall content of the article, thus producing erroneous hits. Consequently, false positives generated both from technical reasons and from conceptual inconsistencies were removed after manual inspection and the search string was accordingly updated with a higher degree of precision (see excluded records in S1 Appendix). In total 184 articles were excluded manually (Fig 1). However, even if reading through the abstracts is an efficient way of excluding false positives, the problem of articles that do not explicitly credit citizen scientists remains a methodological source of error also in the present study, as Cooper et al. have noted [8]. If volunteer contributors are not mentioned in the title, abstract or keywords, they cannot become part of the current dataset. One such example is the article “New Equidistribution Estimates of Zhang Type” by the author Polymath [27]. Here there is no indication whatsoever of this article being the result of a CS project when applying a Topic Search. The only way of detecting this article to conduct an Author Search (AU = “Polymath”). This is also the case with the article by Khatib et al. [28] where the participants of the Foldit CS project are acknowledged as group authors (Foldit Contenders Grp and Foldit Void Crushers Grp). This clearly marks the limit of the Topic Search methodology.

The second dataset attempts a different approach for mapping CS. By scanning a number of review articles that included lists of CS projects as part of their analysis [1,2,8,24,29] it was possible to find 490 unique and valid names of CS projects, which could subsequently be used to construct a search string for the Web of Science (S2 Appendix). This resulted in retrieving 633 articles. Linguistic errors and Keyword Plus false positives were filtered out manually by reading through the abstracts.

## Results

### RQ 1—How has CS developed over time?

**Fig 2:**
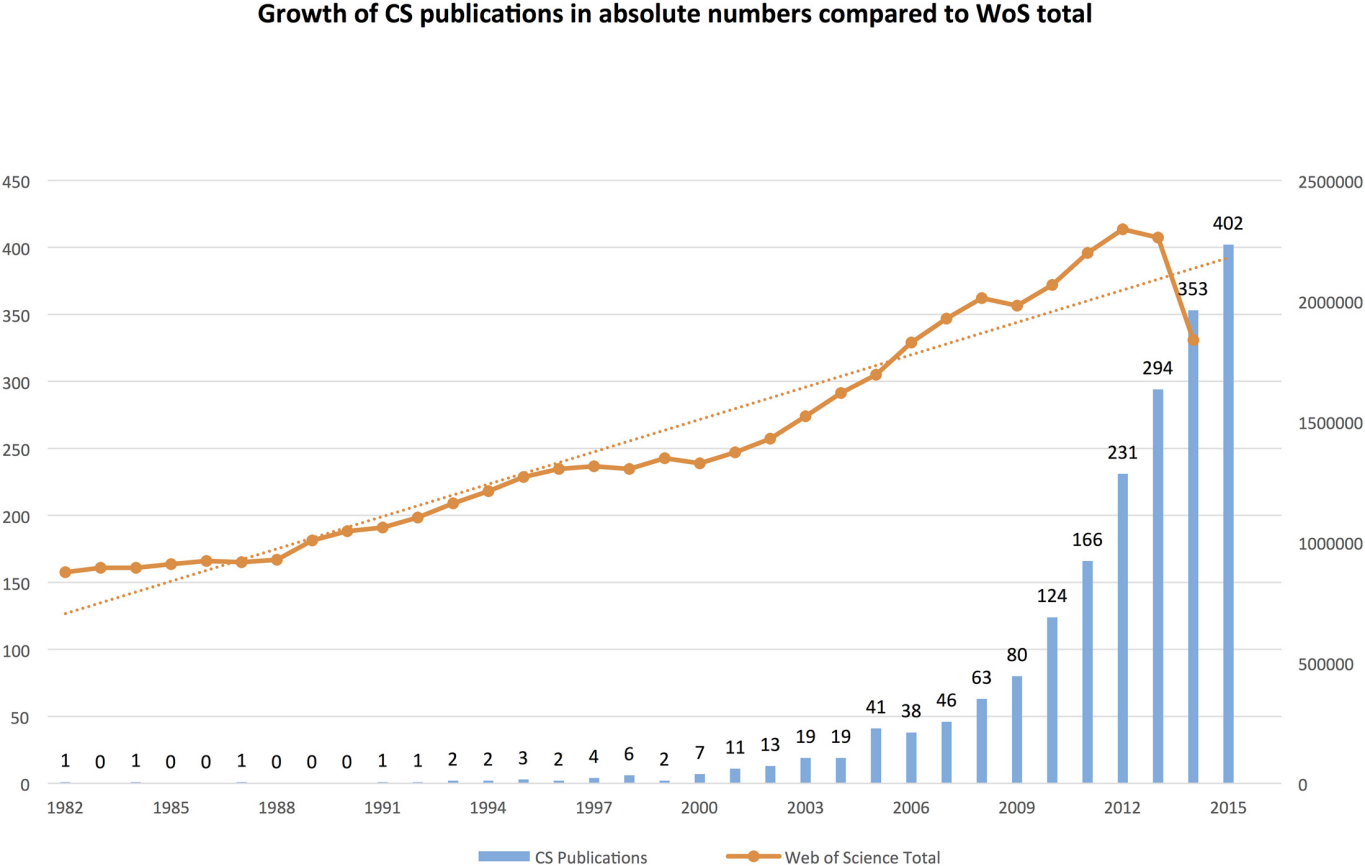
Growth of CS publications compared to WoS total. N = 1935. Search was conducted 2015-12-17 using the search string in S1 Appendix.

The concept and practice of citizen science is barely visible in the WoS in the mid 1990s. Only at the turn of the millennium there is a slow increase. However, around 2010 there is a significant increase in published articles (Fig 2).

**Fig 3:**
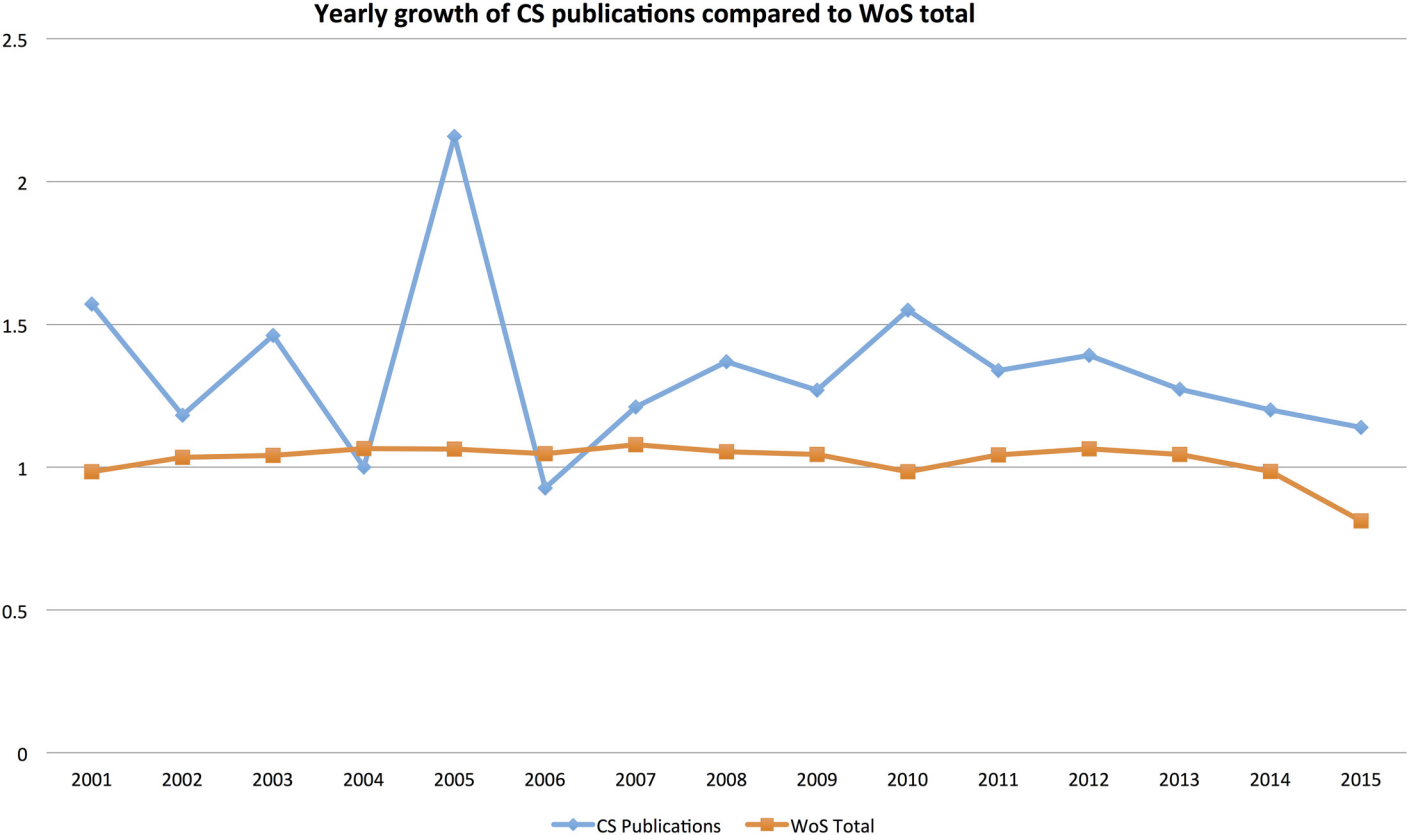
Weighted annual growth of CS publications compared to WoS total. N = 1935. Search was conducted 2015-12-17 using the search string in S1 Appendix. Index value of 1 means that the same amount of articles was produced compared to previous year. Note: the 2015 numbers are incomplete because not all records have yet been added to the database at the time of search.

It is worth noting that the increasing visibility of CS since around 2010 coincides with several digital citizen science projects that use web-platforms for reaching a large crowd of contributors to scientific research, for example Galaxy Zoo, Ebird, FoldIT, Planet Hunters, Genographic Project et cetera (see RQ 3 below). The increase in publications for these projects is further described in the analysis of individual projects below. While the absolute numbers for CS-related publications are low, there is still reason to speak of an emerging trend in relative terms, as shown in Fig 3 where the annual growth of CS articles is compared to the WoS total, showing a stronger development in this particular segment.

### RQ 2—What strands of research have adopted CS?

Article keywords, as defined by authors, were used to draw a map of what types of research that have adopted CS. One method of visualizing the possible connections between various fields of research, or within a research network, utilizes word co-occurences [30] appearing in the meta-data that can be retrieved from the WoS index. In this case we have chosen the author keywords, as they summarize the content of articles accurately. In Table 1 we have listed the most frequent pairs of co-occurring keywords.

**Table 1:** Co-occurence table of the most frequent terms in CS.

Keyword		Co-occurrence
citizen science	monitoring	19
citizen science	climate change	17
citizen science	invasive species	14
nanotechnology	public engagement	14
biodiversity	citizen science	13
crowdsourcing	citizen science	13
citizen science	public participation	11
citizen science	crowdsourcing	10
citizen science	data quality	10
citizen science	phenology	10
public engagement	public understanding of science	10
citizen science	community-based monitoring	9
citizen science	distribution	9
public engagement	science communication	9
volunteered geographic information	crowdsourcing	9
volunteered geographic information	openstreetmap	9
birds	citizen science	8
citizen science	conservation	8
citizen science	public participation in scientific research	8
locally-based monitoring	participatory monitoring	8
neogeography	volunteered geographic information	8
citizen science	survey	7
conservation	citizen science	7
openstreetmap	volunteered geographic information	7
volunteered geographic information	data quality	7
biodiversity monitoring	citizen science	6
citizen science	ciencia ciudadana	6
citizen science	volunteers	6
climate change	citizen science	6
climate change	phenology	6
climate change	public engagement	6
crowdsourcing	volunteered geographic information	6
participatory gis	ppgis	6
science communication	public engagement	6
volunteer monitoring	citizen science	6

(N = 1935, search conducted 2015-12-15). Counts every time a pair of keywords appear in the same article. For a visual representation, see Fig 4.

The keyword “citizen science” is the most common label of CS research. It overlaps frequently with the keywords “monitoring”, “climate change” and “invasive species”, which indicate a proximity with the biology strand of CS-based research. However, it also co-occurs with the notions of “public participation” and “public participation in scientific research”, concepts that belong more to a social science tradition. From geography we also find co-occurrences with keywords such as “crowdsourcing” and “openstreetmap”, which are not restricted to CS research.

To visualize the keywords in Table 1, we used the Gephi (version 0.8.2) software package to create a network map of the co-occurring keywords. As Fig 4 shows, CS-related research can be divided into three main categories. The first and largest one (Blue colour) consists of natural science research on conservation, biodiversity and climate change with one centrally organizing keyword of “citizen science”. The second one (Pink) encompasses Geographical Information Systems research (GIS) centered around the notion of “volunteered geographic information” and the third category (Grey) comprises social scientific research on the phenomenon of participation and science policy gravitating around the keyword “public engagement”.

**Fig 4:**
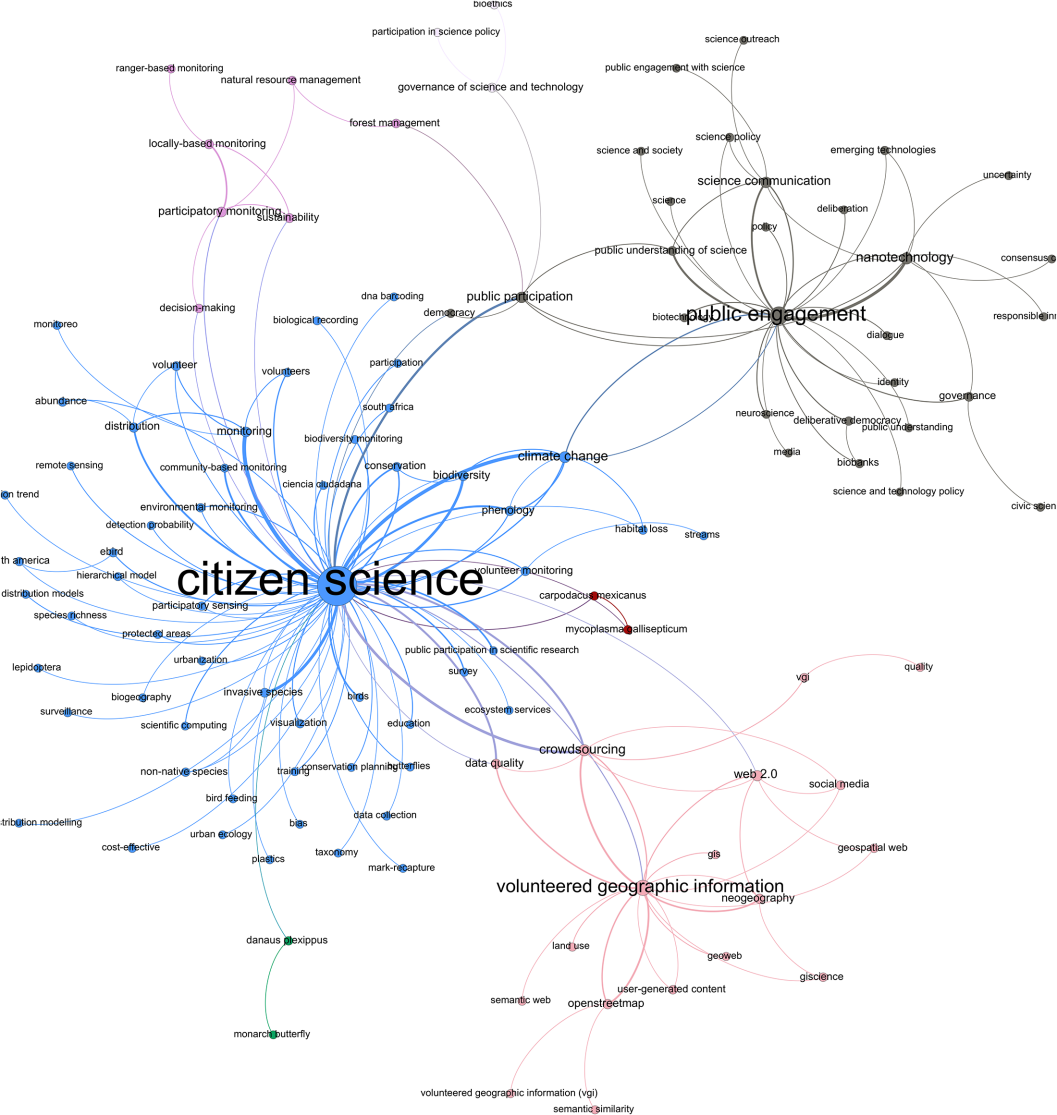
Conceptual structure of contemporary CS. As described by a word co-occurrence network [32] based on keywords (original author keywords, delimited by article title) occurrence network for the search conducted on 2015-12-17 resulting in 1935 hits. To be included, each keyword needed to co-occur at least three times with the same keyword in the dataset. Proximity determined by the ForceAtlas2 algorithm [33] in the software package Gephi (version 0.8.2, http://gephi.org). The larger the node, the more frequently the keyword co-occurs (higher total degree). Colors are selected with the modularity filter for “community detection” [34]. Nodes have been moved slightly to make room for the textual labels.

**Fig 5:**
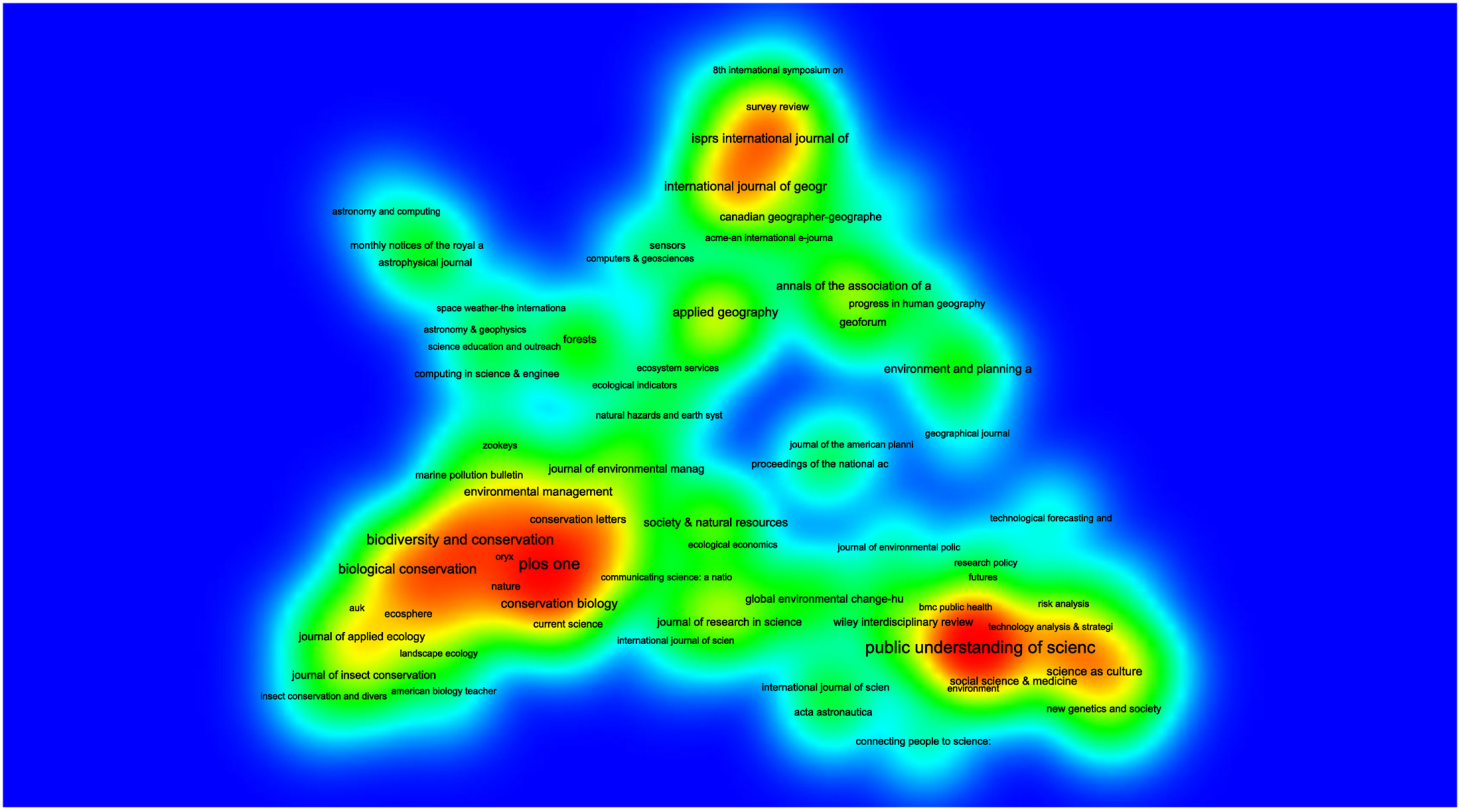
Bibliographic coupling of sources. Generated by VOSviewer [36]. Search conducted 2015-12-17 (S1 Appendix) in the Web of Science Core Collection resulting in 1935 hits. In these 165 sources were identified in cited references (minimum 3 documents of a source). Proximity is determined by bibliographic coupling [35].

The first and most frequent use of citizen science has been carried out under overlapping concepts, such as “community-based monitoring”, “volunteer monitoring” and “participatory monitoring” and is to be found in research on ecology, environmental science, geography and biodiversity conservation (Fig 5). Particularly observation and classification of avian migration (birds and butterflies) is prevalent in this type of research. This is both evident in the keywords “invasive species”, “conservation”, “biodiversity” and “monitoring” (Fig 4) and in terms of journal publications (Fig 5). In quantitative terms, this is the most widely published category of CS.

Furthermore, the fields of Volunteered Geographic Information, and Neogeography are related to each other, especially through various technologies such as “Web 2.0” and “crowdsourcing”. The notions of crowdsourcing and data quality are shared with the cluster around conservation and monitoring

Lastly, concerning the third category of social scientific research on CS, it is imperative to note that most of these studies are conducted by researchers interested in studying the phenomenon of CS, rather than using CS as a method. The central notion here is “public engagement”, which co-occurs frequently with “nanotechnology”. Social scientists are here concerned with the various aspects of participation and democratic involvement and inclusion in science and technology policy, on different aspects of the proliferation of nanotechnology. Moreover, this field also shares the use of the keywords “climate change” and “public participation”. However, what is meant by these terms is not necessarily the same things. When natural scientists use the term public participation, they usually refer to collection of data with the assistance of volunteers, whereas social scientists instead refer to representative engagement of stakeholders in policy processes. These double meanings are sometimes conflated on a policy level and attached with high expectations for the future of CS (see especially the European Commisson Green Paper on Citizen Science [31]).

A number of keywords tie together these three categories. The notions of “crowdsourcing” and “data quality” are shared by the natural sciences and the geographic lines of research, while the notion of “public participation” and “climate change” connects the natural with the social sciences, in broad terms.

**Fig 6:**
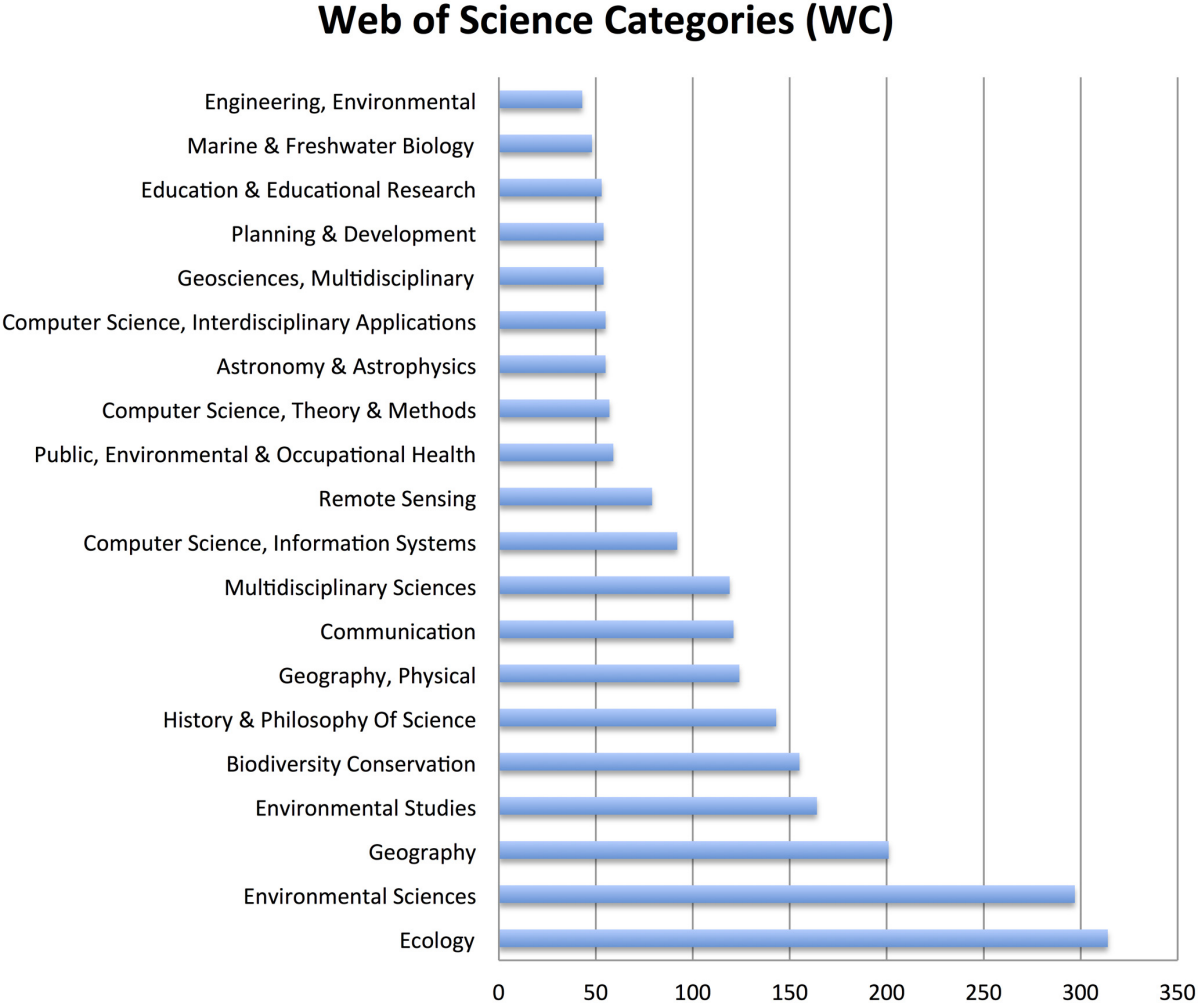
Occurrence of Web of Science Categories. N = 1935, retrieved 2015-12-17. Generated by counting the categories in the WC field of the data extracted from the WoS (S1 Appendix).

In terms of publication patterns, an analysis based on bibliographic coupling [35] of sources reveals a co-citation pattern that is similar to the previously reported in the word co-occurrence network analysis above. As shown in Fig 5 created with the VOSviewer (version 1.5.7) software, the mainstream of journals publishing CS research are to be found in biodiversity, conservation and biological conservation, with the exception of the multidisciplinary PLOS one. Moreover, there is a social scientific cluster with journals such as Public Understanding of Science and Science and Science as Culture, whereas a third cluster is visible in relation to the geographical journals. Thus, there are similarities between the three fields described in the keyword co-occurrence analysis (Fig 4) and the structure of bibliographic coupling (Fig 5). Three distinct fields emerge–natural science, social science and geography–with only sporadic connections between them, both concerning keywords and cited references. Lastly, Fig 5 also renders visible the various astronomical journals that in recent years have had a significant scientific output employing CS. This trend is also evident in Fig 6.

### RQ 3—Which CS projects have a scientific output?

To further describe CS, we conducted a search based on the names of individual CS projects.

Out of 490 projects found, only 78 had a scientific output in terms of publications (see Table 2 for the top projects and S2 Appendix for a list of all projects). The most productive projects were the North American Breeding Bird Survey and similar projects such the Common Birds Census, which also monitor birds, along with the Cooperative Observer Program in which volunteers monitor weather. However, it should be noted that the North American Breeding Bird Survey has conducted yearly surveys since 1967 and has thus accumulated a substantial time series dataset, which consequently generates a high number of articles.

**Table 2:** Scientific output per CS-project, Top 20.

Name	Articles
North American Breeding Bird Survey	178
Galaxy Zoo	88
Common Birds Census	48
Cooperative Observer Program	34
Ebird	32
Nest Record Scheme	20
FoldIT	20
Wetland Bird Survey	17
Genographic Project	16
Planet Hunters	14
Globe at Night	13
Chicago Wilderness	11
North American Amphibian Monitoring Program	9
Evolution Megalab	7
Phytoplankton Monitoring Network	6
NestWatch	6

490 project names were searched in the WoS (For the entire search string and complete data, see S2 Appendix).

As Table 2 shows, there is a clear shift in trends. From the mid 2000s, a number of projects employing digital platforms for observation, collection and processing of data have emerged and have quickly succeed in publishing results in peer-reviewed journals. Notable examples include Galaxy Zoo, Planet Hunters, Globe at Night, Foldit and the Genographic Project. Also Ebird and projects relying on observations performed in the field are increasingly depending on digital platforms for reporting observations and deliver feedback to volunteers.

From the breakthrough of digitally-based CS in the mid 2000s, astronomy (Galaxy Zoo) and bioscience (Foldit) have emerged as fields of research that successfully employ the contributions of non-scientists. Citizen projects in the social sciences and the humanities are, however, absent from the results and not yet developed to a degree that matches those in biodiversity and conservation. This is also the case for medical research.

## Discussion

As our findings reveal, CS and adjacent notions have been used by different fields of research, even though the social sciences, medicine and the humanities are still areas in our data where CS is not utilized to any larger extent, in comparison with the natural sciences and geography. As a general observation, it is foremost in the biological sciences where CS has been adopted as a method with the purpose of collecting observations in the field. The reasons for this has been described as managing problems of time [2], space [37] and large amounts of data that needs a human observer to be classified [3,38]. In addition, the issue of minimizing cost for large-scale observations has been declared as a central reason for implementing CS [1,2,37,39]. In the humanities, the turn towards digital methods (digital humanities) have resulted in large repositories of data the might be eligible for CS-initiatives, for example in projects such as Ancient Lives (http://www.ancientlives.org/) and Operation War Diary (http://www.operationwardiary.org/), which are part of the Zooniverse platform. These projects remain invisible in most scientometric analyses due to their publication patterns, which are often located outside the WoS. In medicine and health, large repositories of data already exist and a strong development of digital mobile technologies, sensors and platforms, spur the data avalanche further on. Currently, medical research has been considered to be less adequate for CS due to ethical concerns and patient security, but there are notable exceptions such as Cell Slider (http://cellslider.net) and Malaria Spot (http://malariaspot.org). Moreover, in the social sciences there has been a long tradition of engaging closely with citizens as *objects* of study, especially in survey-oriented research. However, this cannot be considered to count as instances of CS, since there is no active participation or contribution from the citizens as research *subjects*. Recent calls for developing the social sciences in a CS direction have been proposed, for example Citizen Social Science (http://citizensocialscience.org.uk/) but have not yet made a scientometric footprint and is thus not detectable in our data.

It is a fair speculation that the development of CS will spread to new fields of research in the future, as digital technologies will make large repositories of data possible. Although large data sets pose serious challenges for science, they also promise discoveries if and when resources for their analysis are available. Often the resources necessary include not only equipment, but also expensive human labor, especially if scientists are to be free to perform more demanding conceptual work than routine tasks. Enlisting the help of volunteers is an attractive way for science to expand the workforce needed to work with large data sets. A future challenge here will be to provide a standardized format for sharing meta-data between CS-projects, which will make both sharing of data and evaluation of data quality more accessible. Data quantity, spanning large spatial and time frames can also ensure that even messy data might be reconciled statistically

As Table 2 indicates, the CS projects that have adopted digital platforms for volunteer contributions, (Galaxy Zoo, FoldIt, Ebird) are on the rise in terms of scientific output. However, this effect is exaggerated when compared to historical data because of the increased visibility granted to volunteer contributors in recent years. The emerging digital platforms grant extended possibilities of surveillance of each participant to verify that the contribution is indeed a valid scientific observation, and also, the possibility of having the same classification being verified by a large number of participants before being recorded as data. This marks a both qualitative and quantitative changes in the organization for workload distributions, where citizens can be involved in new instances of the scientific process, and in much larger numbers due to the logistical affordances of digital platforms.

Finally, many CS-projects do not have scientific output as their primary goal. As S2 Appendix shows, 412 of 490 of the CS project surveyed do not have a scientific output. However, it is worth mentioning that publications is but one possible metric. Alternative metrics could be possible by assessing sustainability over time, learning outcomes for participants and the number of volunteers included in the project. In the humanities, the lack of peer-reviewed publications either reflects that many CS projects have other objectives than scientific output as their main goal, or, have failed in terms of quality of data. However, such projects can still produce data that can be incorporated into research. Recently it has been argued that CS-projects in biodiversity related research are underutilized by science concerning data, particularly if they are of a large-scale type covering “large spatial and temporal scales” [29]. In many cases these CS projects are instead targeted to specific issues of concern, initiated by local communities, which may incorporate experts and scientists, but originate outside the academic (or research) institutions and, consequently, most of their funding structures [15,40]. These initiatives emerge from problems identified by communities, often related to environmental issues of pollution, health hazards, species conservation, water and air quality or draining of natural resources [14,22,23]. In such projects the scientific content is often co-produced between professional scientists and citizens, in order to provide evidence in support of campaigns to address the identified issue. However, scientific output (in terms of publications) is typically not aimed for in these circumstances, making them harder to detect via scientometric data. Instead, the main objective usually consists of creating data in order to provide evidence to influence political decision-making or launching legal processes via other publication media, such as blogs and traditional news media. Even though these initiatives mainly emerge from outside of the institutions of science, they heavily rely on scientific standards–and in many cases scientific laboratories–for validating data [15,41]. Even though the motivational factors for participating in CS are under-researched, it is fair to say that there are most likely a multitude of motives, ranging from the personal level to more abstract motivations, such as contributing to science [42] or promoting environmental and societal justice [14,22,23]. This is another argument that highlights the limitations of scientometrics as a method for mapping such a diversified phenomenon, as CS.

## Conclusion

While the concept of CS has gained unprecedented presence in scientific literature during the past decade, the practice itself is much older. Previously, volunteer contributors have not been made visible in scientific articles to a wide extent. However, especially with the introduction of digital platforms, this has changed.

The main field of study employing CS is to be found in biology, ecology and conservation research. Moreover, the social sciences and geography have increasingly started to invite volunteer contributors to research.

In quantitative terms, the largest scientific output is to be found in the fields of ornithology, astronomy, meteorology and microbiology. However, most CS projects fall outside the scope of scientometric evaluation, since scientific output is not a main goal.

## Supporting Information

S1 Appendix Source data for Topic Search dataset (N = 1935). (XLSX)

S2 Appendix Source data for Name Search dataset (N = 633). (XLSX)

## Abbreviations

CS: Citizen science
WoS: Web of Science
3RS: Triple Recursive Search
